# Supplementary material for: Potential of Bacteriocins from Lactobacillus taiwanensis for Producing Bacterial Ghosts as a Next Generation Vaccine
Source: Toxins (Basel). 2020 Jul 1;12(7):432. doi: 10.3390/toxins12070432 (PMC7404994; doi:10.3390/toxins12070432)
Supplement: Supplementary file 1 [file toxins-12-00432-s001.pdf]

Supplementary Materials: Potential of Bacteriocins from *Lactobacillus taiwanensis* for Producing Bacterial Ghosts as A Next Generation Vaccine

Sam Woong Kim, Yeon Jo Ha, Kyu Ho Bang, Seungki Lee, Joo-Hong Yeo, Hee-Sun Yang, Tae-Won Kim, Kyu Pil Lee and Woo Young Bang

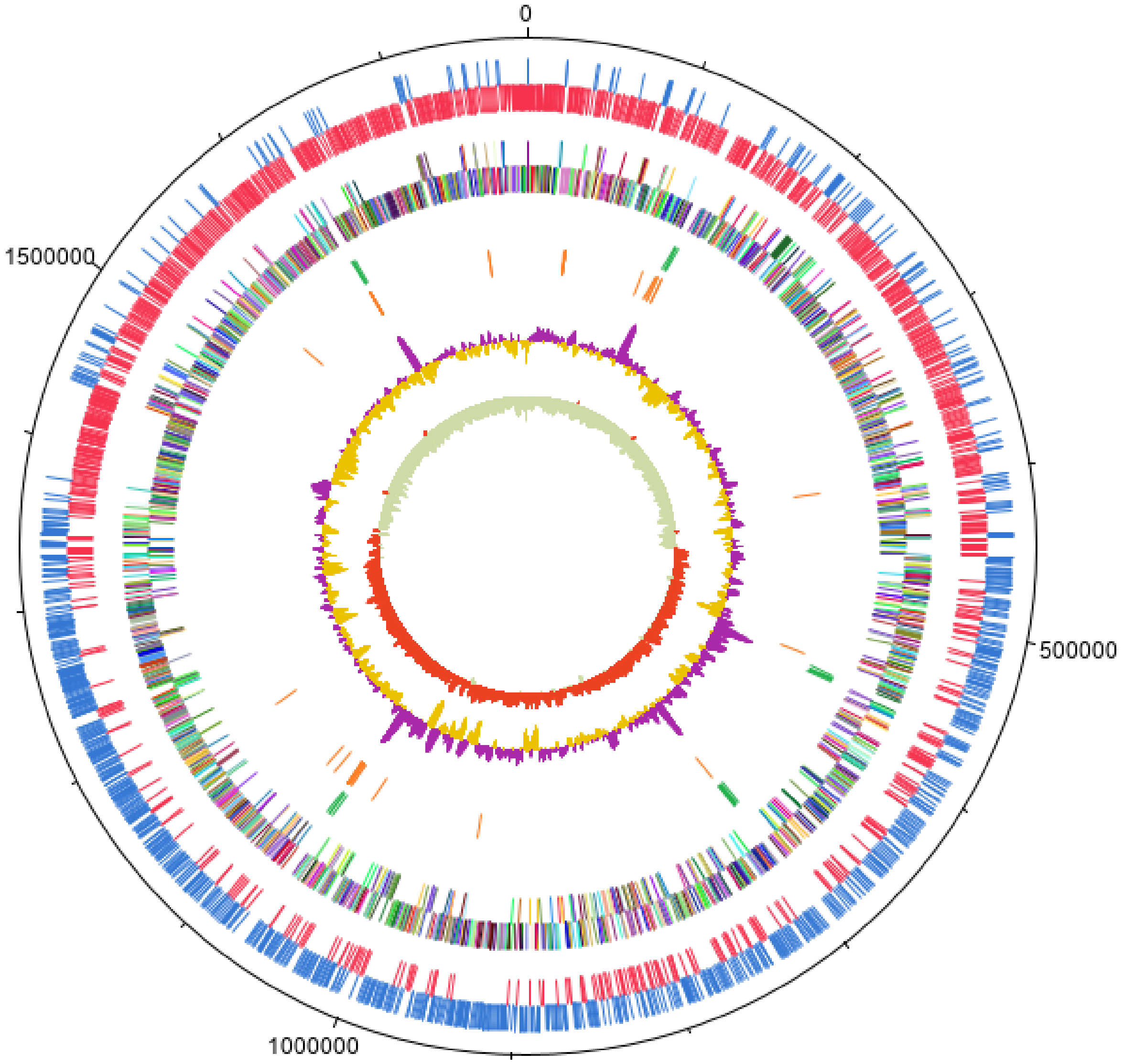

Figure S1. Overall features of the L. taiwanensis genome. The outer scale indicates the coordinates in base pairs. The open reading frames (ORF) is shown on the first two rings; first ring (blue) is the forward ORF and second ring (red) is the reverse ORF. The third and fourth circle shows the ORF which colored by gene annotation; third ring is forward ORF and fourth ring is reverse ORF. The fifth and sixth circle shows rRNA(green) and tRNA genes (orange). The next circle shows the GC content values. Purple and deep yellow colors indicate positive and negative sign, respectively. The inner-most circle shows GC skew, light green indicating negative values whereas deep orange for positive values. The whole-genome sequence data was deposited as Sequence Read Archive (SRA) data in GenBank (SRA No., SRR12004529; BioProject No., PRJNA639099).

Phage holin family protein

Gene name		Compared strain		Compared gene name		
Orf0100 phage holin family protein (HFP)		[Pediococcus pentosaceus (PP)]		phage holin family protein		
**Score**	**Expect**	**Method**	**Identities**	**Positives**	**Gaps**	**Frame**
226 bits (575)	2e-74	Compositional matrix adjust	118/118 (100%)	118/118 (100%)	0/118 (0%)	

```
                   10        20        30        40        50        60
                    |         |         |         |         |         |
Orf01000    MRFWQRVLVNCILFVALTGFFQSNGNFYVSSIWIALVASLILAILNASIRPILQIISLPI
PP HFP      MRFWQRVLVNCILFVALTGFFQSNGNFYVSSIWIALVASLILAILNASIRPILQIISLPI
            ************************************************************
Prim.cons.  MRFWQRVLVNCILFVALTGFFQSNGNFYVSSIWIALVASLILAILNASIRPILQIISLPI

                   70        80        90       100       110
                    |         |         |         |         |
Orf01000     TLLTLGLFSIVINALMLELTSVFVGASNFYFSSFGMTMLISVILSICNTIISNHFTNR
PP HFP      STLLTLGLFSIVINALMLELTVFVGASNFYFSSFGMTMLISVILSICNTIISNHFTNR
            **********************************************************
             TLLTLGLFSIVINALMLELTSVFVGASNFYFSSFGMTMLISVILSICNTIISNHFTNR
```

Holin 1

Gene name		Compared strain			Compared gene name	
Orf01402 holin 1		Pediococcus acidilactici (PA)			holin	
**Score**	**Expect**	**Method**	**Identities**	**Positives**	**Gaps**	**Frame**
250 bits (639)	3e-82	Compositional matrix adjust.	116/172 (67%)	143/172 (83%)	1/172 (0%)	

```
                     10        20        30        40        50        60        70        80        90
                      |         |         |         |         |         |         |         |         |
Orf01402    MQEIYNMTLQMAQSYINKMIQSHAIYGGVIGAMLAIPAWIERDFPTREHGVMIGILLAVLVIDWLTGSQLAKRSPVSEKLSHTANISLIR
PA holin 1  MQDVYYSALRVAQNYISKMGQSHAIYGGVIGGVLAVPAWIESDTPTIEHAVMMAILVLTLFIDWLTGTALARRSPVAERTSHVGNYSIIR
            **:.:*  :*:.:**.**.** ***********.:**:***** * ** **.**:.**: .*.******: **:****:*: **..* *:**
Prim.cons.  MQ22Y222L22AQ2YI2KM2QSHAIYGGVIG22LA2PAWIE2D2PT2EH2VM22IL222L2IDWLTG22LA2RSPV2E22SH22N2S2IR

                    100       110       120       130       140       150       160       170
                      |         |         |         |         |         |         |         |
Orf01402    DFIIVLMCAMAVGLDCVLNTKSFIFAIFTAAFIWQNFYSVLGNLITLGWGKYFPMWMFSLIEKWVQDEVRSKQNKYFPTKGEFHEKIK
PA holin 1  DFIIVAICVMAIGLDYVCKTRSIIFAVFTAAFIWQNFYSVLGNVITLGWDKHFPFWLFNLIKRWVNDEVISKQHKYFP-KGD------
            *************:*****.*:**:*:*.**::**:*** ***:**** **:***** :*.**:*** * :*:*:***:***
Prim.cons.  AFIWQNFYSVLGN2ITLGW2K2FP2W2F2LI22WV2DEV2SKQ2KYFPTKG2FHEKIKDFIIV22C2MA2GLD2V22T2S2IFA2FTA
```

Holin 2

Gene name		Compared strain		Compared gene name		
Orf01553 holin 2		Pediococcus acidilactici (PA)		holin		
**Score**	**Expect**	**Method**	**Identities**	**Positives**	**Gaps**	**Frame**
220 bits (561)	3e-72	Compositional matrix adjust.	110/121 (91%)	117/121 (96%)	0/121 (0%)	

```
                   10        20        30        40        50        60
                    |         |         |         |         |         |
orf01553    MKKISFKNADGSLNGKLIAGIISLLIVLVQQVLAIFGVKFTGDWSAIVGVINTVLTILG
PA holin    MKKISFKNADGSLNGKLIAGIISLLIVLVQQIFAVFGIKFTGDWSAIVAIVNTVLTILG
            *******************************::*:**:***********.::*********
Prim.cons.  MKKISFKNADGSLNGKLIAGIISLLIVLVQQ22A2FG2KFTGDWSAIV222NTVLTILG
                   70        80        90       100       110       120
                    |         |         |         |         |         |
Orf01553    MLGVVTDVQTVTAPTVKSDEESQVEATANKVADEVQAPTSAGAVVNSSKASDTEFTSQASQK
PA holin    MLGVITDVQTVTAPTVKSDEESQVEATANKVADEVQAPTSAGAVVNSSAASETESTSQASQK
            ****:****************************************** **:** ********
Prim.cons.  MLGV2TDVQTVTAPTVKSDEESQVEATANKVADEVQAPTSAGAVVNSS2AS2TE2TSQASQK
```

Holin 3

Gene name		Compared strain		Compared gene name		
Orf01554 holin 3		[Pediococcus pentosaceus (PP)]		holin		
**Score**	**Expect**	**Method**	**Identities**	**Positives**	**Gaps**	**Frame**
169 bits (427)	7e-53	Compositional matrix adjust.	86/87 (99%)	86/87 (99%)	0/87 (0%)	

```
                   10        20        30        40        50        60
                    |         |         |         |         |         |
orf1554     MSQYDDTTKLLMDIQKDVTTTKTKVENIEEKLNQVDDIGNKAEKALAKSIEVEHEIGRIT
PP holin    MSQYDDTTKLLMDIQKDVTTTKTKVENIEEKLNQVDDIGKKAEKALAKSIEVEHEIGRIT
            ***************************************:********************
Prim.cons.  MSQYDDTTKLLMDIQKDVTTTKTKVENIEEKLNQVDDIG2KAEKALAKSIEVEHEIGRIT
                   70        80
                    |         |
Orf1554     QIQNWVIGVLISGVLVTLVIYIAEKFL
PP holin    QIQNWVIGVLISGVLVTLVIYIAEKFL
            ***************************
            QIQNWVIGVLISGVLVTLVIYIAEKFL
```

Figure S2. Comparison of homology for holins. Each holin was compared with the corresponding homolog by NCBI blast (https://blast.ncbi.nim.nih.gov/). The symbols *, : and . indicates the perfect, the strongly similar and the weakly similar alignments, respectively.

Tan 1

Gene name	Compared strain	Compared gene name
Orf00298	L. lactis QU 14	Lacticin Z matured region

Score	Expect	Identities	Positives	Gaps
21.9 bits (45)	1.1	8/18 (44%)	13/18 (72%)	0/18 (0%)

<Total gene comparison>

```
                       10        20        30        40        50
                        |         |         |         |         |
LT LacZ      MAKKVDRNKPLKSLLGMMVVSSLWFAWKNRRKIQEVLNHGQS---------------
LC LacZ      MAGFLKVVQILAK-YGSKAVQ--W-AWANKGKILDWINAGQAIDWVVEKIKQILGIK
             ** :. : * . * .*. * ** *: ** : :* **:
Prim.cons.   MA22222222L22L2G222V2SLWFAW2N22KI2222N2GQ2IDWVVEKIKQILGIK
```

Tan 2

Gene name	Compared strain	Compared gene name
Orf00302	Enterococcus faecium T136	Enterocin B matured region

Score	Expect	Identities	Positives	Gaps
22.7 bits (47)	0.49	10/25 (40%)	14/25 (56%)	0/25 (0%)

<Total gene comparison>

```
                      10        20        30        40        50
                       |         |         |         |         |
Orf00302     ----------MRKNEKGSG----VLEIVGGVLNLAGGALIMAAGIVKLASKTK
Enterocin    ENDHRMPNNLNRPNNLSKGGAKCGAAIAGGLFGIPKGPLAWAAGLANVYSKCN
                        * *: ..*       *.**::.:. *.*  ***:.:: ** :
Prim.cons.   ENDHRMPNNL2R2N2222GGAKC222I2GG222222G2L22AAG22222SK22
```

Tan 3

Gene name	Compared strain	Compared gene name
Orf01552 tan 3	Bacillus brevis	Gramicidin C matured region

Score	Expect	Identities	Positives	Gaps
22.7 bits (47)	1.6	6/14 (43%)	12/14 (86%)	0/14 (0%)

```
<Total gene comparison>
                          10        20        30        40        50        60        70        80
                           |         |         |         |         |         |         |         |
Orf01552 tan 3    MFGSLLQLLGLSRWYHHEQHVDRSNARKASGQSALSYDDFKGLRKLAFWLFVLSIVLNWGFVGAVSLIAWLAMWVVSLLF
Gramicidin C      -------------------------------------------------------------VGALAVVVWLYLWL-----
                                                                               ***::::.** :*:
Prim.cons.        MFGSLLQLLGLSRWYHHEQHVDRSNARKASGQSALSYDDFKGLRKLAFWLFVLSIVLNWGFVGA22222WL22W2VSLLF
```

Figure S3. Comparison of homology for Tans. Each Tan was compared with the corresponding homolog by NCBI blast (https://blast.ncbi.nim.nih.gov/). The symbols *, : and . indicates the perfect, the strongly similar and the weakly similar alignments, respectively.

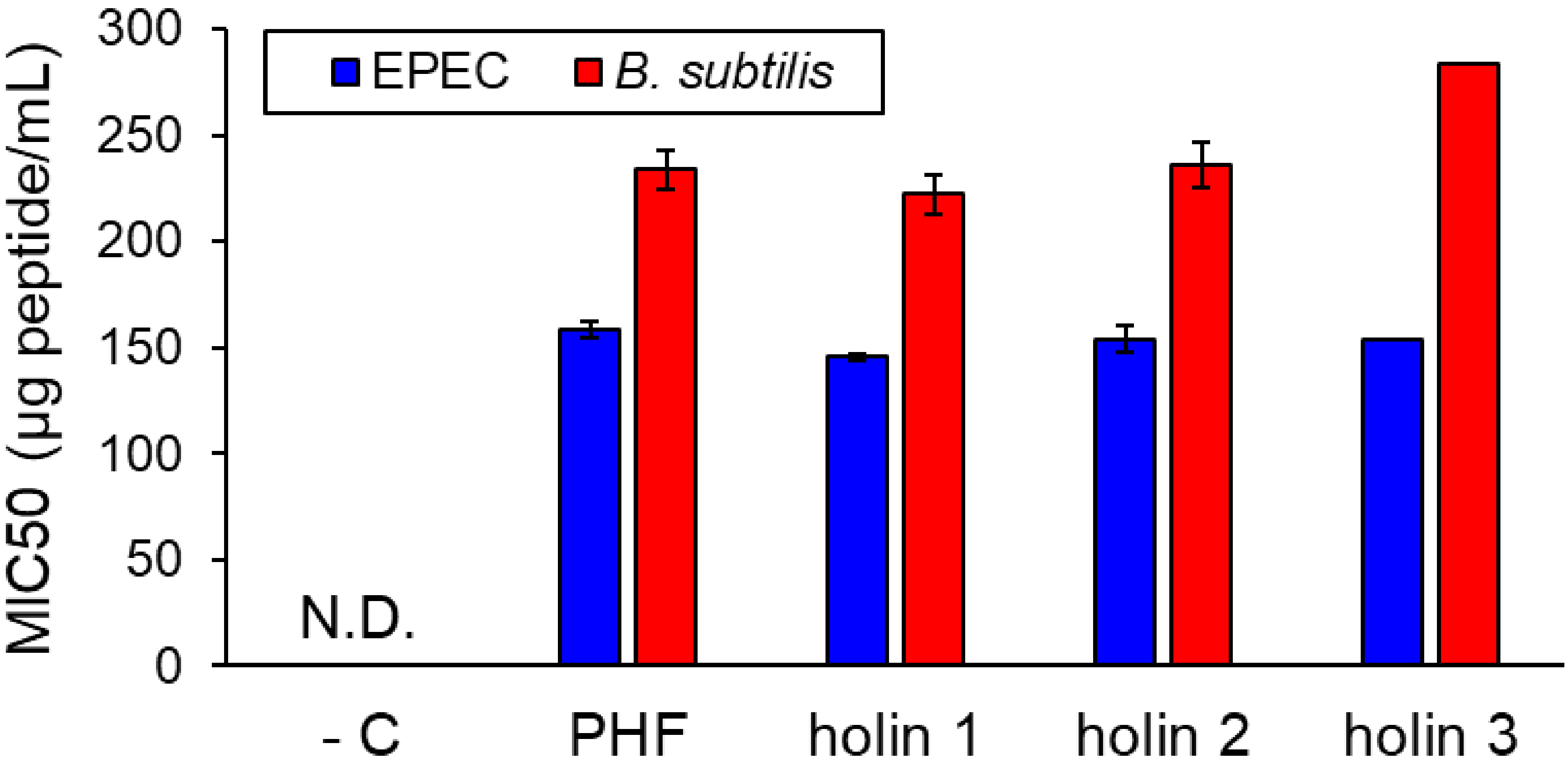

Figure S4. Antibacterial activity of holin homologs identified from *L. taiwanensis* genome. the *E. coli* strains efficiently producing the holin homologs were constructed by transforming *E. coli* TOP10 competent cells with the *holin* genes, *PHF* or *holing 1* to *3*, including their own intact promoter, cloned into the pGEM®-T Easy Vector (Promega, Madison, WI, USA) using primers described in Table S2, and their cell-free supernatant was examined for MIC50 against a Gram-negative bacterium (EPEC) (blue bars) and a Gram-positive bacterium (*B. cereus*) (red bars). The cell-free supernatant from the *E. coli.* Top10 strain, harboring only the plasmid vector without the *holin* genes, was used as a negative control (− C), whose MIC50s against both EPEC and *B. cereus* were not determined (N.D.).

Table S1. Transcriptomic analysis results of *AMPs* from *L. taiwanensis*. *L. taiwanensis AMP* transcripts were examined by RNA-sequencing. L-taiwan-ON and L-taiwan-OD05 indicate total RNA samples extracted from *L. taiwanensis* grown during stationary and exponential phases, respectively.

Test_id	Sample A	Sample B	logFC	Absolute Fold Change	logCPM	*p* Value	FDR	Description	Renamed
orf00298_contig.1.cir	L-taiwan-ON	L-taiwan-ODO5	0.2878146	1.2207896	6.7504428	0.8345538	1	hypothetical protein PEPE_1669 [Pediococcus pentosaceus ATCC 25745]	Taiwanencin 1 (Tan 1)
orf00302_contig.1.cir	L-taiwan-ON	L-taiwan-ODO5	1.3665509	2.5785337	4.3938569	0.3378096	1	hypothetical protein PEPE_1673 [Pediococcus pentosaceus ATCC 25745]	Taiwanencin 2 (Tan 2)
orf01000_contig.1.cir	L-taiwan-ON	L-taiwan-ODO5	0.1665624	1.1223809	6.6363689	0.904152	1	phage holin family protein	phage holin family protein
orf01402_contig.1.cir	L-taiwan-ON	L-taiwan-ODO5	2.6437012	6.2493284	3.9731965	0.0796021	1	holin	holin 1
orf01552_contig.1.cir	L-taiwan-ON	L-taiwan-ODO5	−0.324756	1.2524524	5.8004949	0.8153991	1	uncharacterized protein	Taiwanencin 3 (Tan 3)
orf01553_contig.1.cir	L-taiwan-ON	L-taiwan-ODO5	−2.246791	4.7462601	7.4881531	0.1141369	1	holin	holin 2
orf01554_contig.1.cir	L-taiwan-ON	L-taiwan-ODO5	0.2101973	1.1568464	5.2366903	0.881427	1	holin	holin 3

Table S2. Oligonucleotides used for cloning of the seven *AMP* genes into pGEM®-T Easy Vector.

Gene Name	Forward		Reverse	
	Oligonucleotide Name	Nucleotide Sequence	Oligonucleotide Name	Nucleotide Sequence
PHF	orf01000-NdeI	CATATGTTAGCTTGGTTAATGCCTAG	orf01000-XbaI	TCTAGATAACTCTAAACCTGGACGTG
Holin 1	orf01402-NdeI	CATATGTGACGGCAATACAACCAGCG	orf01402-XbaI	TCTAGACACCTACAATGTACTGGACG
Holin 2	orf01553-NdeI	CATATGGAATGCCAACTTGCGTAATCC	orf01553-XbaI	TCTAGACCAAAAGGACGTGACCACCA
Holin 3	orf01554-NdeI	CATATGCGCCAACAATGGCTGACCAG	orf01554-XbaI	TCTAGATATCGCTGGGTACGTCAGCC
